# Effect of very low-carbohydrate high-fat diet and high-intensity interval training on mental health-related indicators in individuals with excessive weight or obesity

**DOI:** 10.1038/s41598-024-79378-z

**Published:** 2024-11-14

**Authors:** Dominik Sindler, Tomas Dostal, Martina Litschmannova, Peter Hofmann, Lenka Knapova, Laura Maria König, Steriani Elavsky, Lukas Cipryan

**Affiliations:** 1 Department of Human Movement Studies and Human Motion Diagnostic Centre, The University of Ostrava, Ostrava, Czech Republic; 2 Department of Applied Mathematics, Faculty of Electrical Engineering and Computer Science, VSB – Technical University of Ostrava, Ostrava, Czech Republic; 3 Institute of Human Movement Science, Sport and Health, University of Graz, Graz, Austria; 4 Department of Clinical and Health Psychology, Faculty of Psychology, University of Vienna, Vienna, Austria

**Keywords:** Very low-carbohydrate high-fat diet, High-intensity interval training, Mental health, Excessive weight, Obesity, Obesity, Human behaviour, Obesity

## Abstract

Very low carbohydrate high fat (VLCHF) diet and high-intensity interval training (HIIT) are widely utilized for weight reduction and cardiorespiratory fitness improvement, respectively. To assess the acceptability of these approaches, it is essential to examine mental health-related indicators. This secondary analysis of a randomized controlled trial investigated the isolated and synergistic effects of VLCHF and HIIT on mental health-related indicators in individuals with excessive weight or obesity. Sixty-eight participants (age = 42 ± 10.2; 20–60 years; BMI = 29.8 ± 3.7) were analysed across four groups: HIIT (n = 15, 4 males, 11 females), VLCHF (n = 19, 4 males, 15 females), VLCHF + HIIT (n = 19, 4 males, 15 females), and control (n = 15, 4 males, 11 females). The 12-week intervention, involved VLCHF diet or HIIT sessions, depending on group affiliation and completing online questionnaires via Qualtrics software before and after the intervention. The questionnaires included the 12-item Short Form Survey (SF-12) for mental (MHS) and physical health scores (PHS), the Satisfaction with Life Scale (SWLS), and the Perceived Stress Scale (PSS). Using the Kruskal–Wallis test, we found no significant differences in mental health-related indicators between groups after 12 weeks, except for SWLS (p = 0.031; ES = 0.133; medium), which improved significantly in the VLCHF + HIIT group compared to the HIIT group. Our findings indicate that HIIT and VLCHF, alone or combined, do not significantly affect mental health-related indicators.

**Supplementary Information:**

The online version contains supplementary material available at 10.1038/s41598-024-79378-z.

## Introduction

The popularity of carbohydrate restricted diet is rising in recent years as the scientific evidence amounts and available websites or other public information sources indicate^1,2^. A reason for this development is the diet´s high effectiveness for body mass reduction in relatively short time period^3^. Unlike the traditional energy-balanced model, where a caloric deficit causes weight loss, a significant reduction in carbohydrates leads to a decrease in the insulin-glucagon ratio, an increase in lipolysis and fat oxidation without changing energy intake, leading to weight loss goals^4^. The very low-carbohydrate high-fat diet (VLCHF), in particular, is defined by the amount of carbohydrate intake of 20–50 g/d or 10% of daily energy intake and has been shown to result in clinically meaningful weight loss^5^. There is also evidence that VLCHF diet can be considered effective to treating obesity and type 2 diabetes, and as a prevention or treatment option for other of civilisation diseases such as type 1 diabetes, non-alcoholic fatty liver disease, neurodegenerative diseases and cancer^6,7^.

High-intensity interval training (HIIT) is ranked in the top 20 of the ACSM Fitness Trends rankings for 2023 and 2024, declaring its current popularity among exercise approaches^8^. HIIT is defined as short exercise intervals (mostly lasting from 60 to 240 s) at an intensity of between 80%-100% peak heart rate or aerobic capacity interspersed with resting periods^9^. HIIT has a positive effect on aerobic capacity and performance and is a popular training method among athletes due to its time efficiency and comparable effect to high-volume training^10–12^. Due to its safety and efficacy in cardiometabolic health, this training method provides additional health benefits for inactive or individuals excessive weight or obesity, such as improving blood pressure or cholesterol control, reducing body fat, or may even have a positive impact on mental health components^13–17^.

VLCHF diet and HIIT have been shown to have a positive effect on physical health and together could be used as an effective tool to reduce the prevalence of obesity^18^. Not only weight control but also cardiovascular or cardiorespiratory factors seem to play a crucial role in the prevention of obesity and its related diseases, which are potentially positively influenced by VLCHF diets or HIIT^19–21^. There is a promising outlook on how the combination of these dietary and exercise strategies may lead to optimal health outcomes concerning body composition and cardiorespiratory fitness^22^.

The World Health Organization defines health as “a state of complete physical, mental and social well-being and not merely the absence of disease or infirmity”^23^. Therefore, health can be viewed as a complex system comprising multiple components that interact with one another^24^ emphasizing the significance of considering all these components to attain optimal health. Health status is one of the determinants of overall quality of life (QoL) and a major determinant of health-related quality of life (HRQoL)^25^. HRQoL is a subset of overall QoL that describes physical, psychological and social well-being and is the most commonly used indicator of treatment success or failure^26^. Indeed, psychological or mental well-being plays an important role in achieving positive weight loss outcomes in populations with excessive weight or obesity, as confirmed in a study by Alhalel et al.^27^, where significantly greater weight loss was achieved in those with improved or stable mental health over 12 months. Examining indicators of mental well-being when assessing interventions aimed at modifying weight-related behaviours is therefore of utmost importance.

HIIT may have positive effects on mental health indicators among healthy inactive individuals^15^ but also in populations with excessive weight or obesity and these benefits are similar to those of conventional high-volume training^28,29^.However, little is known about the effect of the VLCHF diet on mental health and there is a lack of evidence on its effect on indicators such as life satisfaction or perceived stress, as well as the synergistic effect of the VLCHF diet and HIIT on these indicators in individuals with excessive weight or obesity. Drawing upon data obtained from a randomized controlled clinical trial which has previously showcased the efficacy of VLCHF and HIIT in altering body composition, cardiometabolic risk indicators, and cardiorespiratory fitness^22,30^ including lipidomic and metabolomic alterations^31^, the primary objective of this research was to conduct an initial examination of the potential isolated and synergistic effects of the VLCHF regimen and HIIT on mental health-related indicators among individuals grappling with excessive weight and obesity. Based on our previous results with this dietary approach^32^, we hypothesize that the observed mental health-related indicators will not be significantly different in the intervention groups compared to the control group.

## Results

### Sample

The flow chart of participants dropout and reasons is presented in Fig. 2. Sixty-eight participants were included in the analysis, while detailed baseline characteristics are presented in Table 1. There were no differences between groups at baseline across all descriptive outcomes.

**Table 1:** Baseline characteristics of participants. Legend: BMI, body mass index; WHtR, waist-to-height ratio; SWLS, satisfaction with life scale; PSS, perceived stress scale; PHS, physical health score; MHS, mental health score; Md (95% CI), median and 95% confident interval.

	HIIT (n = 15)	VLCHF (n = 19)	VLCHF + HIIT (n = 19)	Control (n = 15)	Between group differences
	Md (95%CI)	Md (95%CI)	Md (95%CI)	Md (95%CI)	p-value
Male:Female	4:11	4:15	4:15	4:11	
Age (year)	46 (38.53; 53.11)	43.00 (34.39; 46.84)	43.00 (33.08; 52.31)	40.00 (34.18; 51.57)	0.697
Height (cm)	168.00 (161.05; 173.92)	168.40 (164.45; 173.75)	169.50 (161.40; 177.17)	171.90 (161.81; 176.92)	0.964
BMI	28.73 (26.90; 30.36)	29.69 (27.36; 31.91)	30.82 (26.78; 34.32)	28.04 (26.18; 33.07)	0.685
WHtR (score)	0.59 (0.54; 0.62)	0.60 (0.56; 0.64)	0.63 (0.56; 0.68)	0.59 (0.53; 0.63)	0.676
SWLS	5.60 (4.84; 6.20)	5.60 (4.28; 6.00)	4.50 (4.00; 5.32)	5.60 (5.04; 5.96)	0.134
PSS	1.10 (0.59; 1.58)	0.90 (0.77; 1.33)	1.60 (1.20; 1.93)	1.10 (0.92; 1.30)	0.021^a^
PHS	53.71 (52.09; 55.50)	54.21 (51.69; 55.90)	52.74 (48.36; 56.78)	55.28 (52.45; 57.12)	0.715
MHS	53.89 (49.29; 57.74)	55.23 (53.21; 56.69)	50.36 (41.26; 53.76)	53.94 (47.65; 55.87)	0.081

Kruskal- Wallis test: ^a^Significant difference between group at baseline (p < 0.05) caused be difference between VLCHF + HIIT and Control groups.

### Mental health-related indicators

There were no significant differences between the study groups in mental health-related indicators at baseline, except for PSS that differed by study group (p = 0.021; ES = 0.146; medium). The difference was caused by the differences between VLCHF + HIIT and the VLCHF group (see Table 1), with the VLCHF group reporting lower chronic stress scores.

**Fig. 1:**
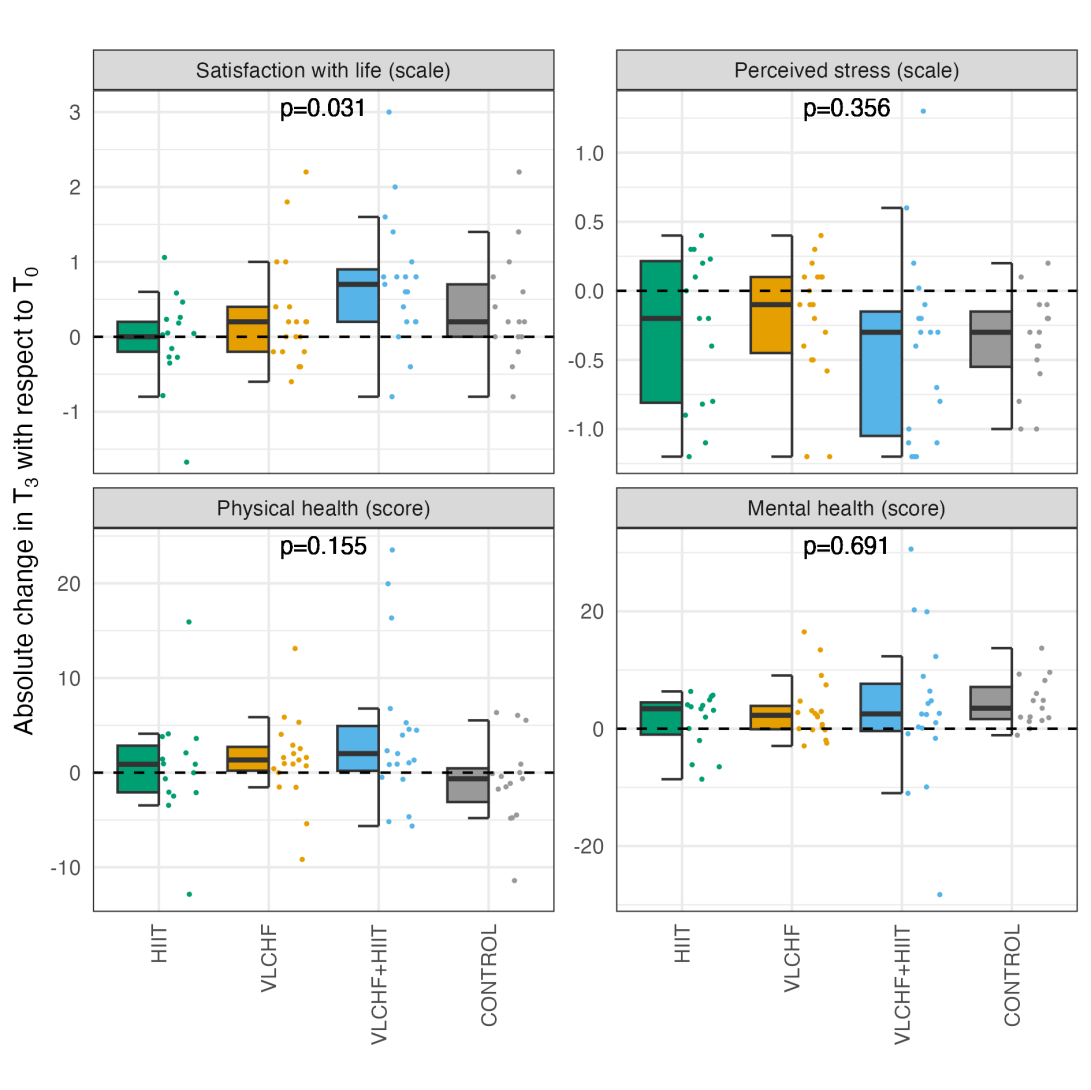
Absolute changes in SWLS, PSS, PHS and MHS after 12 weeks (T_3_) with respect to baseline (T_0_).

Statistically significant between group differences after 12-weeks intervention were found only for SWLS (p = 0.031; ES = 0.133; medium). Post-hoc analysis revealed a significant difference between HIIT and VLCHF + HIIT groups, with the combined condition resulting in higher life satisfaction. Other variables did not differ significantly between groups after the 12-week intervention (see Table 2). Since the analysed sample size is smaller than in the original study, we also present intra and inter-individual differences in Fig. 1.

**Table 2:** Mental health-related indicators differences after 12 weeks. Legend: SWLS, satisfaction with life score; PSS, perceived stress score; PHS, physical health score; MHS, mental health score.

	HIIT (n = 15)	VLCHF (n = 19)	VLCHF + HIIT (n = 19)	Control (n = 15)	Between group differences Kruskal–Wallis test
	ΔMd (95% CI)	ΔMd (95%CI)	ΔMd (95%CI)	ΔMd (95%CI)	p-value
SWLS	0.00 (-0.50; 0.40)	0.20 (-0.10; 0.80)	0.70 (0.40; 1.10)*	0.20 (-0.10; 1.00)	0.031^b^
PSS	-0.20 (-0.65; 0.10)	-0.10 (-0.45; 0.00)	-0.30 (-0.75; − 0.14)*	-0.30 (-0.60; -0.15)*	0.356
PHS	0.88 (-2.11; 2.95)	1.34 (-0.30; 3.29)	2.01 (0.18; 7.93)*	-0.65 (-3.13; 2.42)	0.155
MHS	3.40 (-1.58; 4.57)	2.28 (0.31; 5.91)*	2.52 (-0.86; 9.54)	3.49 (1.95; 7.48)*	0.691

Data are presented as the median differences (ΔMd) between baseline minus 12-week measures with 95% confidence interval (CI).

Two–tailed Wilcoxon signed–rank test: *significant within group differences (p < 0.05) for baseline vs. 12–week.

Kruskal- Wallis test: Post–hoc analysis: ^b^Significant between group difference was found between HIIT and VLCHF + HIIT.

## Discussion

In this secondary analysis of a 12-week randomized controlled trial, we found no significant differences of mental health-related indicators between the VLCHF diet or HIIT (or their combination) compared to the control group in individuals with excessive weight and obesity. Recently, we have shown improvements in cardiorespiratory fitness, body composition, and cardiometabolic risk factor management in parental studies^22,30,31^, which together with the current results suggest promising health benefits of these lifestyle interventions.

### Satisfaction with life

We found significant improvements in SWLS post intervention (r = 0.736; large) (see Supplementary Fig. S1 online) in the VLCHF + HIIT which was reflected in the comparison with HIIT group, however these changes were not statistically significant compared to the control group. There is evidence that life satisfaction is correlated with body composition and exercise levels at all ages of individuals with excessive weight and obesity^33–35^; the potential boost in SWLS might also contribute to greater improvements in physical health. Given that we did not find significant differences in any of the intervention variants compared to controls, it could be assumed that changes of parameters related to mental health require a longer period to meaningfully change, especially if assessed generally. One could anticipate more robust effects on more proximal outcomes such as specific emotions or mood states, which have been shown to mediate the effects of both diet and physical activity or exercise on life satisfaction, be it directly or indirectly through its impact on self-control or adherence. Indeed, satisfaction with life, as measured with SWLS, has been shown to be relatively stable^36^ suggesting that this examined variable may be less sensitive to changes than, for example, changes in mood and emotion. Furthermore, it is important to note that SWLS was somewhat lower in the VLCHF + HIIT group at baseline, compared to the three other groups, although the difference was not statistically significant.

### Perceived stress

The VLCHF + HIIT group reported significantly higher PSS at baseline compared to the other groups and perceived stress level as a function of the intervention (r = -0.554; large) significantly decreased. However, this decrease was not significantly different from the control group which, also reported decreases in perceived stress post intervention (r = -0.778; large) (see Supplementary Fig. S2 online). A similar lack of effects of HIIT on perceived stress was previously reported in university students^37^ or in testicular cancer patients^38^. In contrast, a reduction in the stress component was found with a longer 10-month intervention in inactive women with obesity^39^. Furthermore, adding a dietary intervention component to an exercise intervention was shown to lower stress, confirming prior research in postmenopausal women with excessive weight/obesity^40^. It remains to be determined whether the effect for lowering perceived stress is reflected also in physiological stress markers. A study in healthy men suggested that both exercise and diet positively influenced physiological stress, but no synergistic effects of the two interventions were found^41^. That study however tested effects of a whole foods diet, not the VLCHF diet. Further research is needed to examine the combined and independent effects of VLCHF and HIIT on both perceived and physiological stress.

### Physical and mental health

We found no significant changes in PHS or MHS between groups after 12 weeks. In addition, no significant changes in perceived physical health were found for any intervention group except VLCHF + HIIT (r = 0.480; medium) after 12 weeks (see Supplementary Fig. S3 online). Regarding perceived mental health, participants in the VLCHF diet (r = 0.588; large) and control group (r = 0.873; large) showed improvements after 12 weeks (see Supplementary Fig. S4 online). Prior studies on the effects of low carbohydrates diets on physical and mental health reported inconclusive results. For instance, Cohen et al.^42^ reported that a ketogenic diet led to significant improvements in physical, but not mental, health in women with ovarian or endometrial cancer. In contrast, Yancy et al.^43^, who targeted healthy participants with excessive weight found that although there was significant improvement in physical health score in both the ketogenic and high-carbohydrate low-fat diet groups, mental health score improved only in the ketogenic diet group after the 24-week intervention. In the literature, HIIT also appears to have a positive effect on physical or mental health indicators, with 3 days per week of HIIT in healthy individuals associated with significant improvements physical and mental health score compared to a non-active control group^15^. Since we previously found positive changes in objectively measured physical health indicators in VLCHF and VLCHF + HIIT or HIIT in the context of body weight reduction or improvement in exercise capacity^22^, it is interesting to observe that individuals might not have perceived the improvements to a similar extent. For this reason, our results are inconsistent with previously mentioned literature showing that objective improvements in health status associated with VLCHF diet or HIIT among clinical, healthy or individuals with excessive weight/obesity, are related to subjective ratings of these physical or mental health indicators.

### Strengths and limitations

To the best of our knowledge, this is the first randomized controlled clinical trial that investigated the effects of VLCHF diet on life satisfaction or perceived stress and also assessed the synergistic effects of HIIT and VLCHF regarding mental health and thus addresses an important research gap^32^. However, some important limitations of the present study need to be acknowledged. First, additional participants had to be excluded from the analysis due to a lack of completed questionnaires or missing data. Due to this fact, the calculated sample size estimation in the parent study, i.e., 76 people to detect a large effect, was not reached. This investigation therefore should only be considered a first test, also of potential negative side-effects, and (preregistered) replications in larger samples are warranted. Second, despite randomization, the baseline median PSS was significantly higher in the VLCHF + HIIT group, which may have been also related to the smaller sample size. Third, although we did not find negative group differences within and between interventions, some participants answered opposite to the study conclusions (see Supplementary Fig. S1, S2, S3, S4). On the other hand, these isolated changes were in all study groups. Fourth, the control group improved significantly over time in some of the monitored variables, although they received no dietary or exercise interventions, and despite being asked not to increase their physical activity, and only participating in laboratory measurements at the beginning and end of the intervention. These changes in participants’ well-being could be a result of participating in research, a phenomenon known in behavioural science as the Hawthorne effect^44^. Another possible explanation could be that participants had to report their diet daily to the mobile app, which in itself could be seen as a self-monitoring intervention. Changes in individuals’ emotions, cognitions or behaviour due to being measured in a study is known as the measurement reactivity effect^45^, and it has been previously demonstrated for the digital measurement of several health behaviours including physical activity^46^ and self-reported consequences of an intervention^47^. Still, it is important to note that the design of the study followed recommendations for reducing reactivity effects in clinical trials, such as not varying measurement protocols between trial arms^48^, which has likely contributed to reducing the effect to a minimum.

## Conclusion

High-intensity interval training and a very low-carbohydrate high-fat diet, or a combination of these, have no significant effect on the mental health-related indicators measured. This study provides one of the first insights into the synergistic effects of this type of diet and exercise intervention on mental health. However, further studies with larger sample sizes focusing on more variables related to mental health or social indicators are needed to better understand the overall impact of these lifestyle changes on the health of individuals with excessive weight or obesity.

## Methods

### Parent clinical trial

This is a secondary data analysis of a randomized, controlled, four-arm trial with parallel exercise and/or dietary intervention (ClinicalTrials.gov: NCT03934476). A total of 91 participants were randomly assigned to one of four groups: 1) HIIT and usual diet, 2) VLCHF diet and usual physical activity (no regular exercise), 3) VLCHF diet and HIIT, and 4) control group (usual diet and physical activity without regular exercise). The intervention took place over 12 weeks. Data to assess body composition and cardiorespiratory fitness were collected using dual-energy X-ray absorptiometry (DXA; Hologic Discovery A, Waltham, MA, USA), and graded exercise testing to voluntary exhaustion using the Balke-Ware treadmill protocol^49^. Blood samples were collected and blood pressure was measured to identify cardiometabolic risk factors. Previous publications reported on the effect of VLCHF and HIIT on body composition and cardiorespiratory fitness levels^22^, cardiometabolic risk factors^30^ and lipidomic and metabolomic profiles in individuals with excessive weight and obesity^31^.

### Participants

**Fig. 2:**
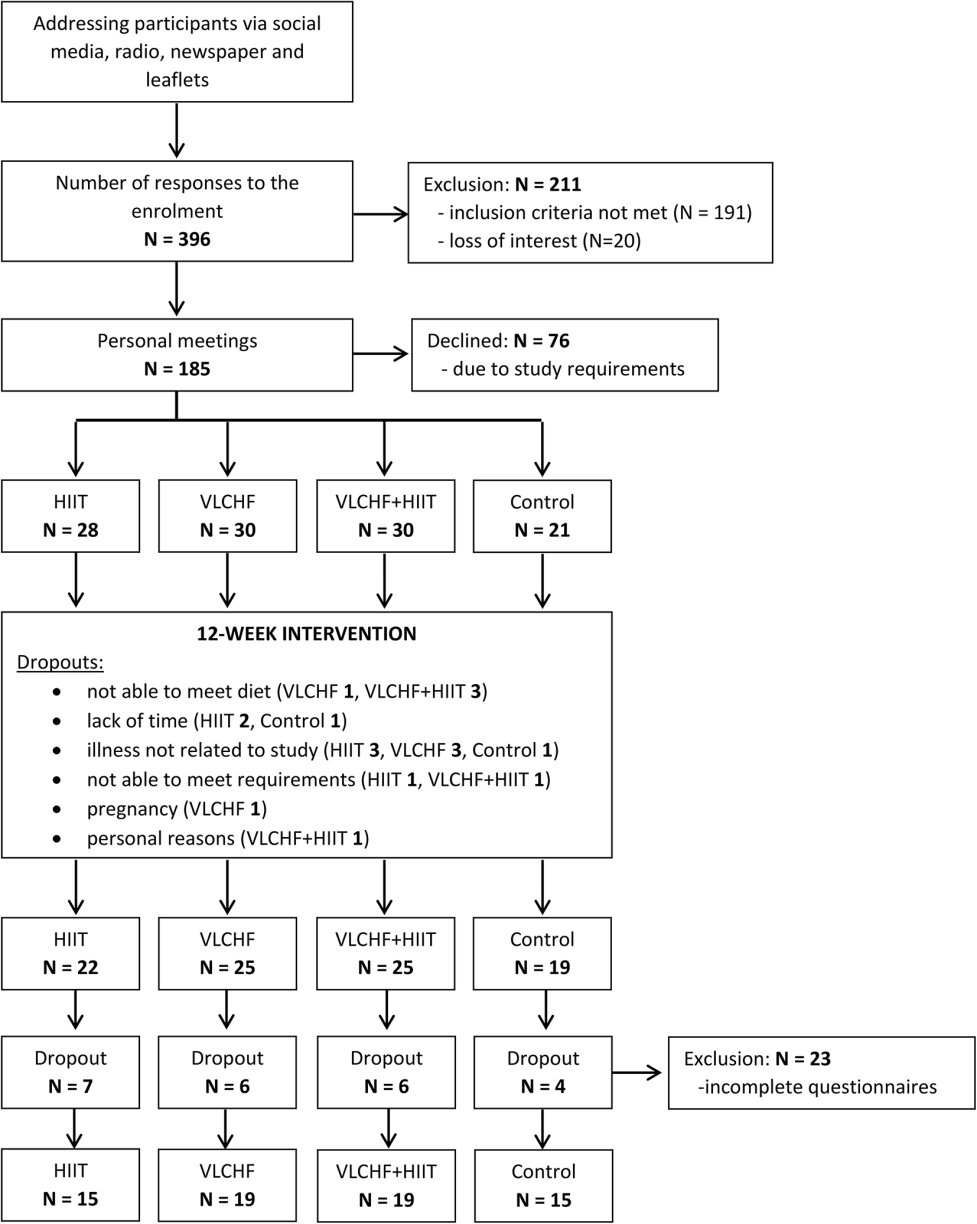
Study flow chart.

The inclusion criteria were: age between 20 and 59 years, non-smokers, excessive weight or obesity (BMI 25–40 kg/m^2^), no specific sports training or regular exercise (low level of physical activity), no excessive alcohol intake, willingness to accept random assignment, overall good health status without any indications of physical or mental diseases, no pregnancy or breast-feeding, not adhering to any diet plan, passing the Physical Activity Readiness Questionnaire (PAR-Q), body mass stable for the last 2 months before the intervention (< 5% of total body mass), not on any medications connected to psychological health or that affect body weight or energy expenditure and no previous experience with VLCHF diet or HIIT. A detailed description of the inclusion and exclusion criteria is presented elsewhere^22^. To be included, participants had to have complete questionnaire data at the beginning and end of the intervention (weeks 0 and 12); those who did not meet this added criterion were excluded (n = 23). Out of the 91 participants who underwent the 12-week intervention, 68 were included into analysis (see Fig. 2 for the participant flow including sample sizes of the four groups). Written informed consent was obtained from all participants and the study design was approved by the ethics committee of the University of Ostrava. All methods were performed in accordance with the relevant guidelines and regulations.

### Intervention

#### High-intensity interval training

At the start of the study, participants in the HIIT and VLCF + HIIT groups were given detailed verbal and written information about the HIIT program. Participants were asked to self-perform 3 HIIT sessions per week for the 12-week intervention, with one session at the laboratory visit at weeks 4, 8 and 12 being a laboratory-assessed exercise test and the remaining two sessions self-performed at home. Each HIIT had a 5-min slow walking warm-up and cool-down phase. The main phase of the HIIT consisted of a 1:1 ratio of a 3-min high-intensity walking interval (RPE 18–19 on the Borg scale) followed by a 3-min low-intensity walking interval (RPE 9–11). High intensity intervals were increased by 4, 6 and 8 intervals per training session in the first, second and third four-week periods. The total high intensity training time increased to 12, 18 and 24 min after each four-week period, with total training times of 31, 43 and 55 min, respectively. Training intensity was measured by a Polar M430 heart rate monitor watch (Polar M430; Polar Electro, Oy, Finland) and data were continuously uploaded to the Polar flow mobile app. The data were analysed regularly to monitor adherence. Participants were reminded to record any additional training sessions in addition to the study protocol.

#### Very low-carbohydrate high-fat diet

The VLCHF diet was defined as not exceeding the intake of 50 g of carbohydrate (CHO) per day^50^. No specific calorie or energy goal was set, but participants in these intervention groups were advised to compensate for the reduction in energy intake caused by restricting CHO intake by increasing their intake of natural non-trans fats. The protein intake was recommended as 1.5g/kg lean body mass and all sweetened and grain-based products should be minimized. Before and during the intervention, participants received nutritional advice from a dietitian on the recommended diet structure (on request or at least once a month) and a handbook with a food list, guidelines for calculating macronutrient amounts, and recipe suggestions. Alcoholic beverages were prohibited during the intervention as well as dietary supplements for 1 month before and during the intervention. Caffeinated beverages were restricted before laboratory sessions only.

### Procedure

Participants completed 4 laboratory sessions at baseline, week 4, week 8, and week 12 to determine body composition, perform a graded exercise test, measure blood pressure and draw blood (baseline, week 4 and week 12), with the exception of the control group who participated only at baseline and week 12. Participants in the HIIT and control groups were advised not to change their usual diet and the control group was also advised not to increase their exercise habits. The control group was not given any dietary advice nor intervention devices. All four groups recorded their food intake daily throughout the study, starting 7 days before the start of the intervention, in an online application (www.kaloricketabulky.cz). Participants in all four groups completed online questionnaires daily, weekly, and monthly for 12 weeks of the intervention to assess quality of sleep, positive and negative affect, acute stress, resilience, pain, fatigue, anxiety and depression. Questionnaires were sent to respondents’ email addresses using Qualtrics software (Qualtrics, versions 2018, 2019, Provo, UT, USA. https://www.qualtrics.com).

### Mental health-related indicators

#### Physical and mental health

Physical (PHS) and mental health (MHS) were assessed using the 12-item short form of the health survey (SF-12). Several different response scales and options were used, depending on the nature of the construct to be assessed^51^. It was scored separately as mental health and physical health scores according to the manual^52^.

#### Satisfaction with life

The Satisfaction with Life Scale (SWLS) contained 5 items and used a response scale reflecting the level of agreement with the statements, with 1 being “strongly disagree” and 7 being “strongly agree”^53^. The SWLS score was calculated as the mean of all responses.

#### Perceived stress

The Perceived Stress Scale (PSS) consisted of 10 questions that participants answered on a scale of 0–4, with a response number of 0 being “never” and 4 being “very often”^54^. Items 4, 5, 7 and 8 were inversely coded before the scale mean was calculated for the PSS score.

### Statistical analyses

Scores for the SWLS, PSS and SF-12 were calculated using IBM SPSS statistics 24 software (IBM SPSS Statistics for Windows, version 24.0. Armonk, NY. https://www.ibm.com/spss). Statistical analyses were performed using R, a free software environment for statistical computing and graphics (R Core Team (2021), version 4.1.2. https://www.R-project.org/).

Mental health-related indicators, as measured by SF-12, SWLS and PSS, was described by the median and 95% confidence interval at two time points at baseline (T_0_) and after 12 weeks (T_3_). Subsequently, absolute changes in the observed variables at T_3_ compared to T_0_ were analysed. Due to deviations from normality as indicated by Shapiro–Wilk tests, nonparametric tests were used. Differences between groups at baseline were tested using Kruskal–Wallis test.

Changes over time within groups were tested using Wilcoxon signed-rank tests for each outcome. The effect size (ES) of the observed changes was determined using Wilcoxon’s effect size (r) test including its 95% confidence interval. The thresholds for ES were 0.10 to < 0.30 (small), 0.30 to < 0.50 (medium), and ≥ 0.50 (large).

Finally, absolute changes in the variables were compared between the HIIT, VLCHF, HIIT + VLCHF and control groups using the Kruskal–Wallis test at T_0_ and T_3_. The Dunn test was used to analyse specific pairs of samples for stochastic dominance. Dunn’s test multiple comparison p-values was adjusted with the Benjamini–Hochberg method. The effect size of the observed differences was assessed using the eta-squared based on the H-statistic, including its 95% confidence interval. The thresholds for the ES eta square were 0.01 to < 0.08 (small), 0.08 to < 0.26 (medium), and ≥ 0.26 (large)^55^.

For all analyses, statistical significance was set at p < 0.05.

## Supplementary Information

Supplementary Material 1.

Supplementary Material 2.

Supplementary Material 3.

Supplementary Material 4.

## Data Availability

The raw data supporting the conclusions of this article will be available upon request pending application and approval from a corresponding author, without undue reservation.
