# Supplementary figures and images for: Effect of very low-carbohydrate high-fat diet and high-intensity interval training on mental health-related indicators in individuals with excessive weight or obesity
Source: Sci Rep. 2024 Nov 14;14:28023. doi: 10.1038/s41598-024-79378-z (PMC11564516; doi:10.1038/s41598-024-79378-z)

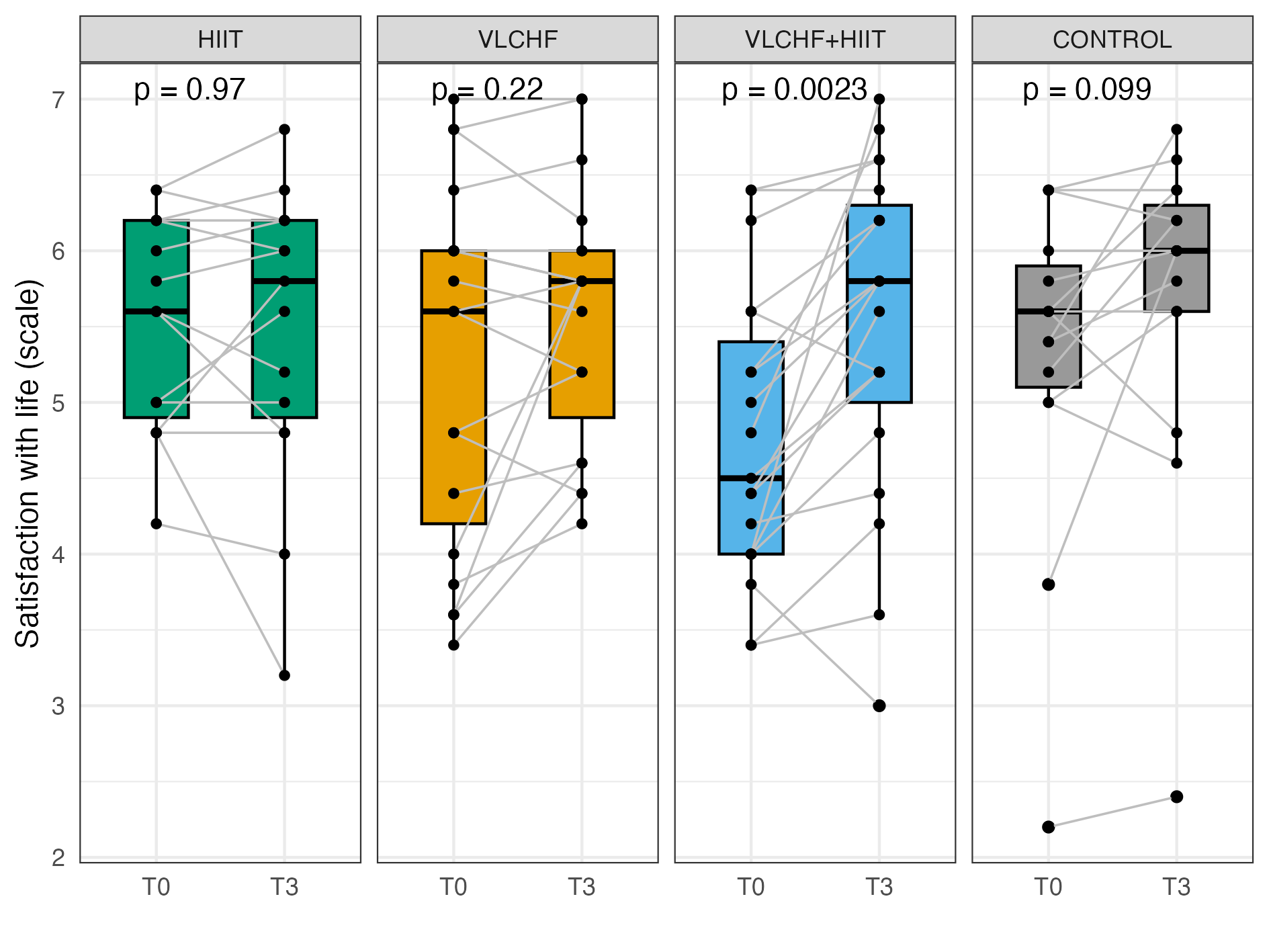

Supplement: Supplementary file 1 — Supplementary Material 1. [file 41598_2024_79378_MOESM1_ESM.png]

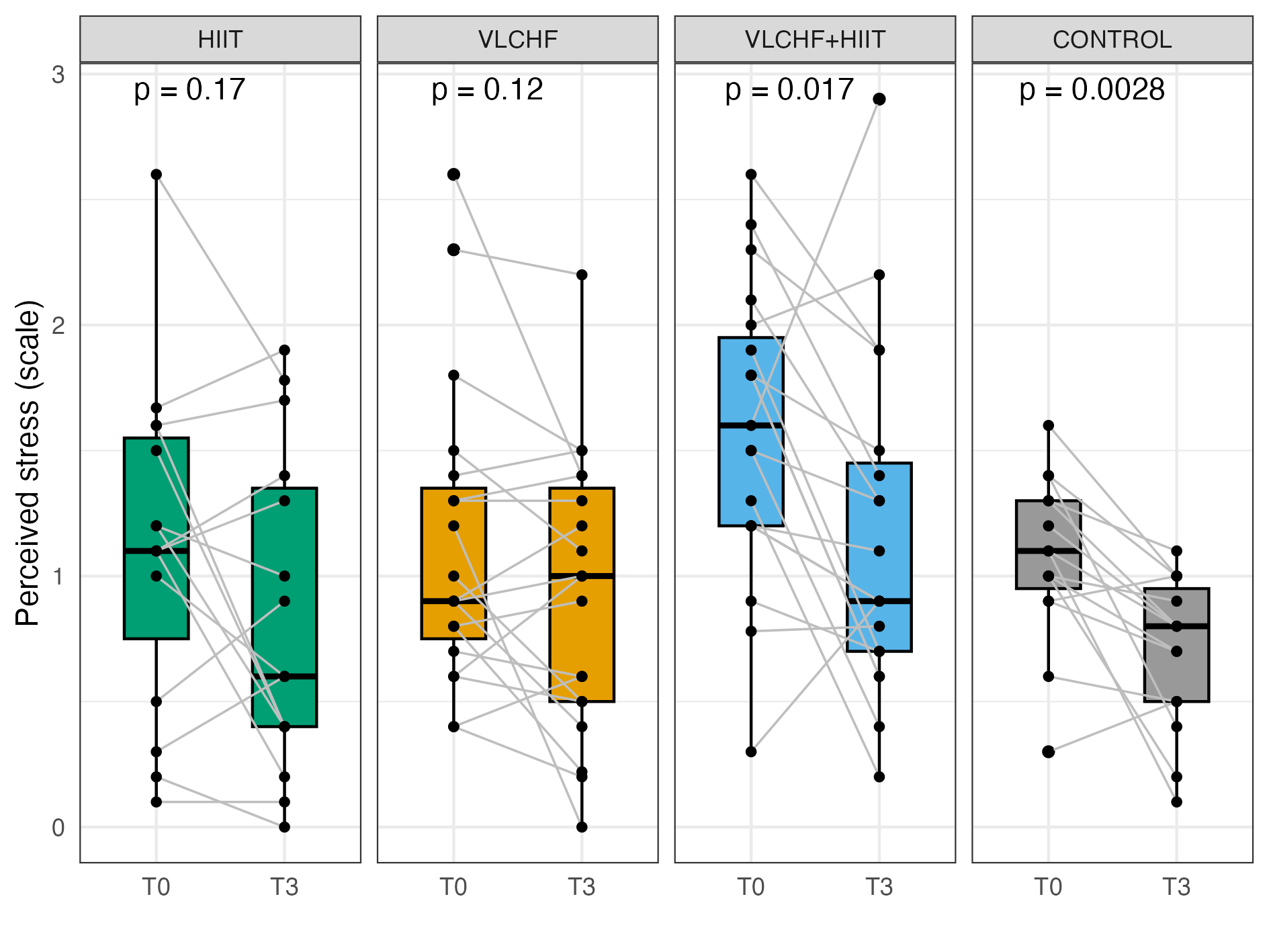

Supplement: Supplementary file 2 — Supplementary Material 2. [file 41598_2024_79378_MOESM2_ESM.png]

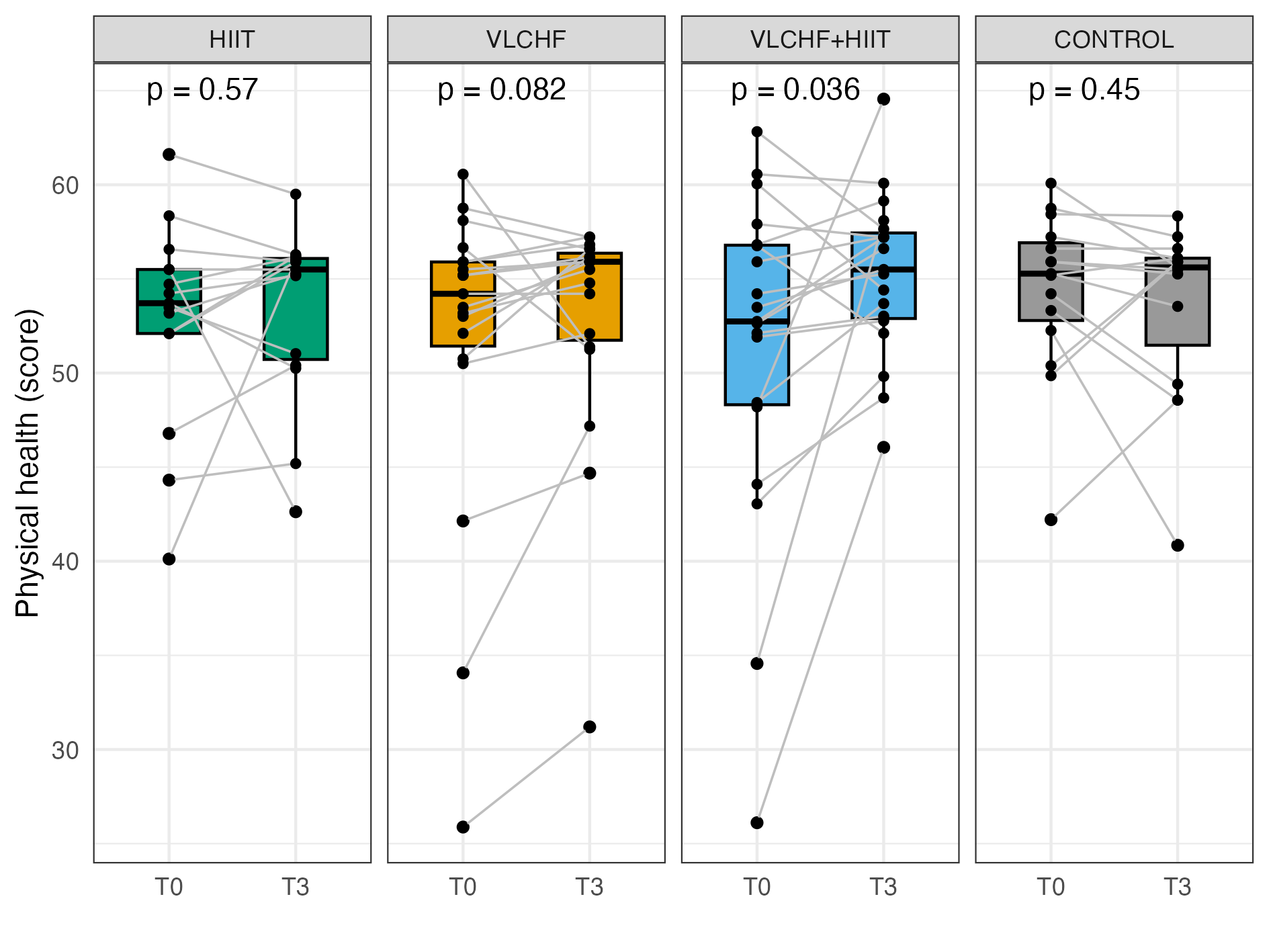

Supplement: Supplementary file 3 — Supplementary Material 3. [file 41598_2024_79378_MOESM3_ESM.png]

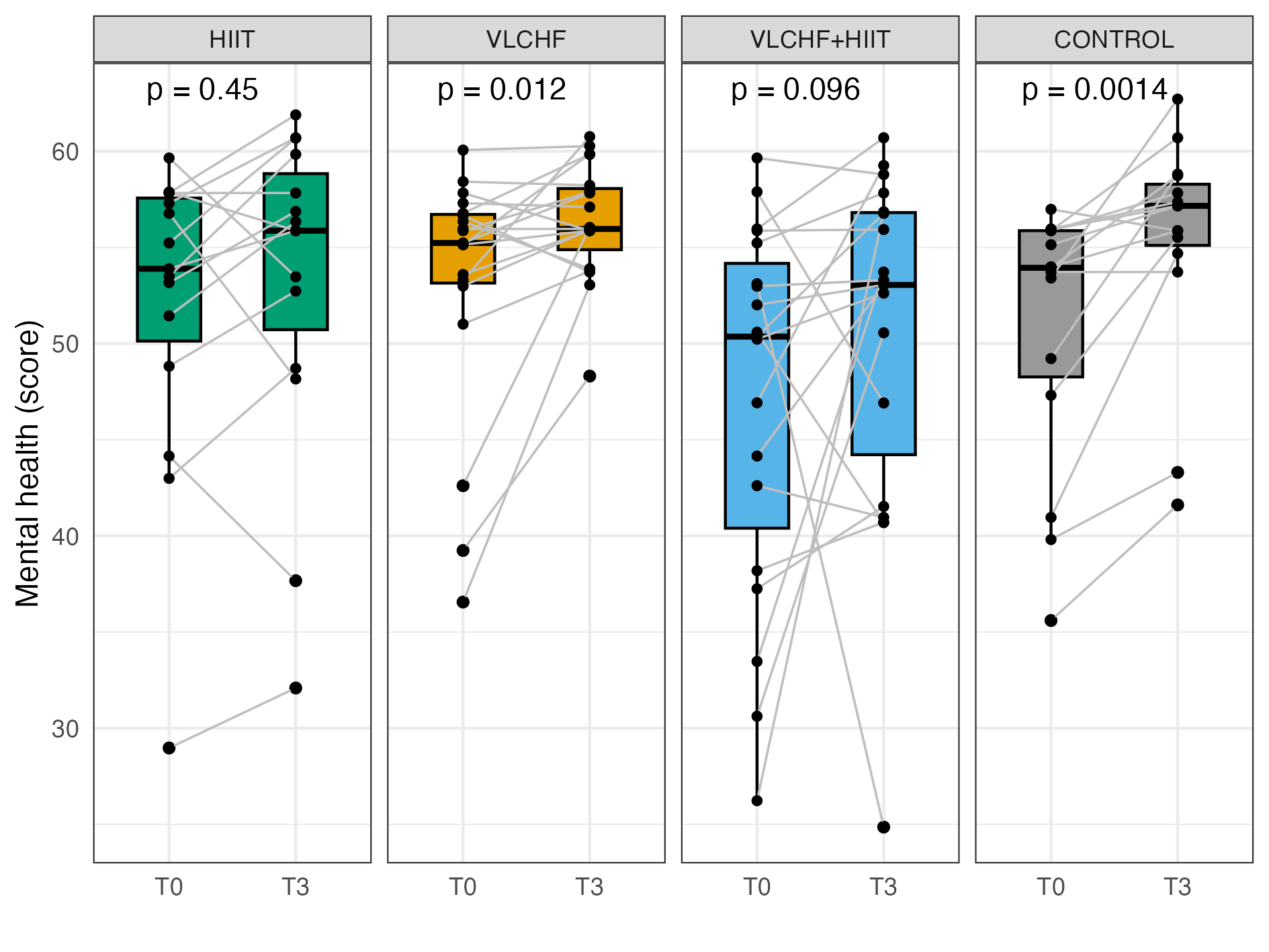

Supplement: Supplementary file 4 — Supplementary Material 4. [file 41598_2024_79378_MOESM4_ESM.png]
